# Supplementary material for: A phylogenomic approach reveals a low somatic mutation rate in a long-lived plant
Source: Proc Biol Sci. 2020 Mar 11;287(1922):20192364. doi: 10.1098/rspb.2019.2364 (PMC7126060; doi:10.1098/rspb.2019.2364)
Supplement: Supplementary analyses [file rspb20192364supp1.docx]

**Supplementary Information**

**1. Effect of biological replicate filters on variant calling**

In our original positive control analysis (see main manuscript), we required all three biological replicates (consisting of independently extracted and sequenced samples from three neighbouring leaves on a single branch) from a single branch to have the same genotype in order for that site in that site to be used in downstream analyses. We did this to minimise the false positive mutation calls, because we hypothesised when planning the study that false positive mutation calls would be severely detrimental to our ability to perform the positive control. During the review process we were asked to quantify how many false-positive mutation calls were eliminated by requiring all three genotypes to agree.

To do this, we repeated the “variant calling for positive control” part of our analysis described in the main body of the manuscript twice. In the first analysis, we required just two replicates to agree, i.e. we retained all sites where at least two genotypes were identical. In the second analysis we performed a simple majority rule analysis. That is, we retained, as in the first analysis, all sites where at least two genotypes were identical. We additionally retained all sites in which just one of the three genotypes was called (i.e. we excluded sites in which there were two genotypes called but the genotypes differed, and those in which three genotypes were called but all three differed from one another). We call this the ‘majority rule’ analysis below.

The results of these analyses are shown in supplementary table 1.1, with the results from the original analysis shown for comparison.

| Supplementary table 1.1: the effects of the replicate filter on the detection of variants. | | | |
| --- | --- | --- | --- |
| **Filter** | **Mutations called** | **False Negative Rate** | **False Discovery Rate (Number of false positive mutations)** |
| All 3 replicates must agree (main ms) | 99 | 77.3% | 56.3% (55.71) |
| At least 2 replicates must agree | 335,867 | 68% | 97.5% (327,351) |
| Majority rule for any number of replicates | 335,867 | 68% | 97.5% (327,351) |

We note that the results from the majority rule filter are identical to those from the filter where at least two replicates must agree - this is because sites at which just a single replicate was genotyped are removed by other filters in our pipeline.

These additional analyses suggest that the use of three replicates was vital to the success of our positive control analysis. Requiring all biological replicates to have matching genotypes produced a somewhat higher false negative rate (77.3% versus 68%) than less stringent approaches, but a dramatically lower false discovery rate (56.3%) than either of the less stringent filters (97.5%). Of note - the number of false positive mutations described in supplementary table 1 for the analysis when all three replicates must match (55.71 false positive mutations on average over 100 replicates) is much higher than the false positive rate in our full Maximum Likelihood analysis using DeNovoGear described in the main manuscript (0.11 false positive mutations on average over 100 replicates). This is expected - the analysis we report above is based on the positive control analysis in the main manuscript, which uses a relatively crude replicate filter that only retains sites at which all three replicates were genotyped and the genotypes agree. In contrast, the full Maximum Likelihood model implemented in DeNovoGear that we describe in the second part of the manuscript leverages the information contained in the biological replicates more effectively than those that use the GATK pipeline. The point of the GATK analysis was to provide a positive control on which to base downstream analyses.

For the positive control analyses in which we used the replicate filter, the numbers in table 1.1 show the importance of using three replicate filters. For example, our phylogenetic positive control would be extremely unlikely to be informative if we used an alignment of 335,867 mutations (as recovered when we require only two genotypes to agree) of which 327,351 are likely to be false positives, simply because the phylogenetic signal from the 2.5% of the data which are true mutations is likely to be swamped by the 97.5% of the data which are false positives. On the other hand, a dataset of 99 variable sites of which roughly 56 are false positives (and thus roughly 43 are likely to be true positives) has a much better chance of recovering the expected phylogenetic tree.

**2. False negative rates estimated for each branch of the tree**

To estimate the false negative rate for the whole dataset, we simulated 14,000 in-silico somatic mutations (1000 on each branch), and used our DeNovoGear pipeline (described in the section of the methods entitled “Variant calling for estimating the rate and spectrum of somatic mutations”) to measure how many of these in-silico mutations we could recover. From these data it is also possible to examine whether our false negative rate differs among branches, simply by counting for each branch how many of the 1000 simulated mutations were correctly recovered by our pipeline. These data are provided in supplementary table 2.1.

| Supplementary Table 2.1: False negative rate in each branch of the tree | | |
| --- | --- | --- |
| **Branch Mutated** | **Mutations Recovered from 1000 simulated** | **False negative rate** |
| E->F | 317 | 68.3% |
| D->1 | 301 | 69.9% |
| D->2 | 285 | 71.5% |
| C->3 | 276 | 72.4% |
| B->4 | 306 | 69.4% |
| E->5 | 307 | 69.3% |
| G->6 | 263 | 73.7% |
| G->7 | 234 | 76.6% |
| A->E | 328 | 67.2% |
| F->G | 316 | 68.4% |
| A->B | 306 | 69.4% |
| F->8 | 281 | 71.9% |
| C->D | 323 | 67.7% |
| B->C | 350 | 65.0% |

The false negative rates range from 65.0% to 76.6%. The standard deviation is 2.96%. One value is more than two standard deviations away from the mean (76.6% false negative rate in branch G->7, which is higher than the mean plus two standard deviations which is 75.96%), suggesting that our ability to infer mutations on the branch that leads from node G to branch tip 7 is slightly lower than would be expected from the distribution of false negative rates. This is most likely explained by the lower coverage of sequencing data for sample 7. Of the 8 samples, the mean sequencing coverage at sites that had a coverage of at least 1 sequenced base (i.e. excluding all sites which had no coverage) were: Sample 1, 42.6x; Sample 2, 38.8x; Sample 3, 37.2x; Sample 4, 39.5x; Sample 5, 46.1x; Sample 6, 35.3x; Sample 7, 25.9x; Sample 8, 29.2x. Sample 7 had the lowest coverage (25.9x) of all sequenced samples. Indeed, sequencing coverage appears to explain roughly 90% of the variation in the false negative rate among samples: the Spearman’s ρ of the correlation between the false negative rate of the 8 tip branches and their sequence coverage is -0.90.

**3. Effects of the ExcessHet filter on variant calling**

In our analysis we use the ExcessHet filter to remove sites from the analysis in an attempt to reduce the false positive rate. The motivation for this was that we suspected we would often see false positive mutations at sites which were actually heterozygous in all samples, but at which genotyping errors might have caused a false-positive mutation to be called. A reviewer asked us to investigate the effect of removing this filter, so we repeated our analysis without it. As expected, removing the filter increases the number of mutations detected to 520, suggesting that applying this filter excluded 430 putative mutations. Re-calculating the false negative rate and the number of false positives for this data suggest that the false negative rate is 80.3%, and the number of false positive mutations is 286.78 (implying a false discovery rate of 55.2%). The slight increase in the false negative rate (80.3% without the ExcessHet filter, vs. 77.3% with it) was unexpected - naively one expects that filtering fewer variants should lead to a *lower* false negative rate. Further examination of the data suggests that this effect occurs because the ExcessHet filter removes a number of small and likely spurious indels (specifically, it removes 643,383 small indels). Removing the filter therefore leads to a small number of variants near these likely spurious indels being removed by our indel proximity filter, leading to an increased false negative rate when not using the ExcessHet filter. The large increase in the number of false positive mutations is expected - it implies that when not using the ExcessHet filter roughly half of the inferred mutations are false positives. The inclusion of these false positives in our analysis would make many of our downstream analyses more problematic, and would make it particularly difficult to judge whether any given mutation was of biological origin or a technical artefact.

**4. Correlation between physical and genetic branch lengths**

Supplementary figure 4.1 shows a plot of the linear model forced through the origin that relates the physical branch length in meters to the genetic branch length in inferred somatic mutations, as reported in the main manuscript. This plot shows a significant positive relationship such that longer physical branches tend to have more mutations (R^2^ = 0.82, p<0.001). The plot was forced through the origin because by definition, a physical branch of length 0 cannot have accumulated somatic mutations. To check whether forcing the origin of the plot through zero is reasonable for this data, we repeated the regression without forcing the origin through zero, allowing us to check whether the uncertainty of the intercept includes zero. This analysis confirms that the uncertainty on the intercept includes zero: the intercept is estimated as 3.19, with 95% confidence intervals of -0.10 to 6.48. Figure 4.2 shows a plot of the linear model that is not forced through the origin. This analysis also shows a significant positive relationship such that longer physical branches tend to have more mutations, but as expected the strength of the relationship is weaker (R^2^ = 0.38, p<0.025).


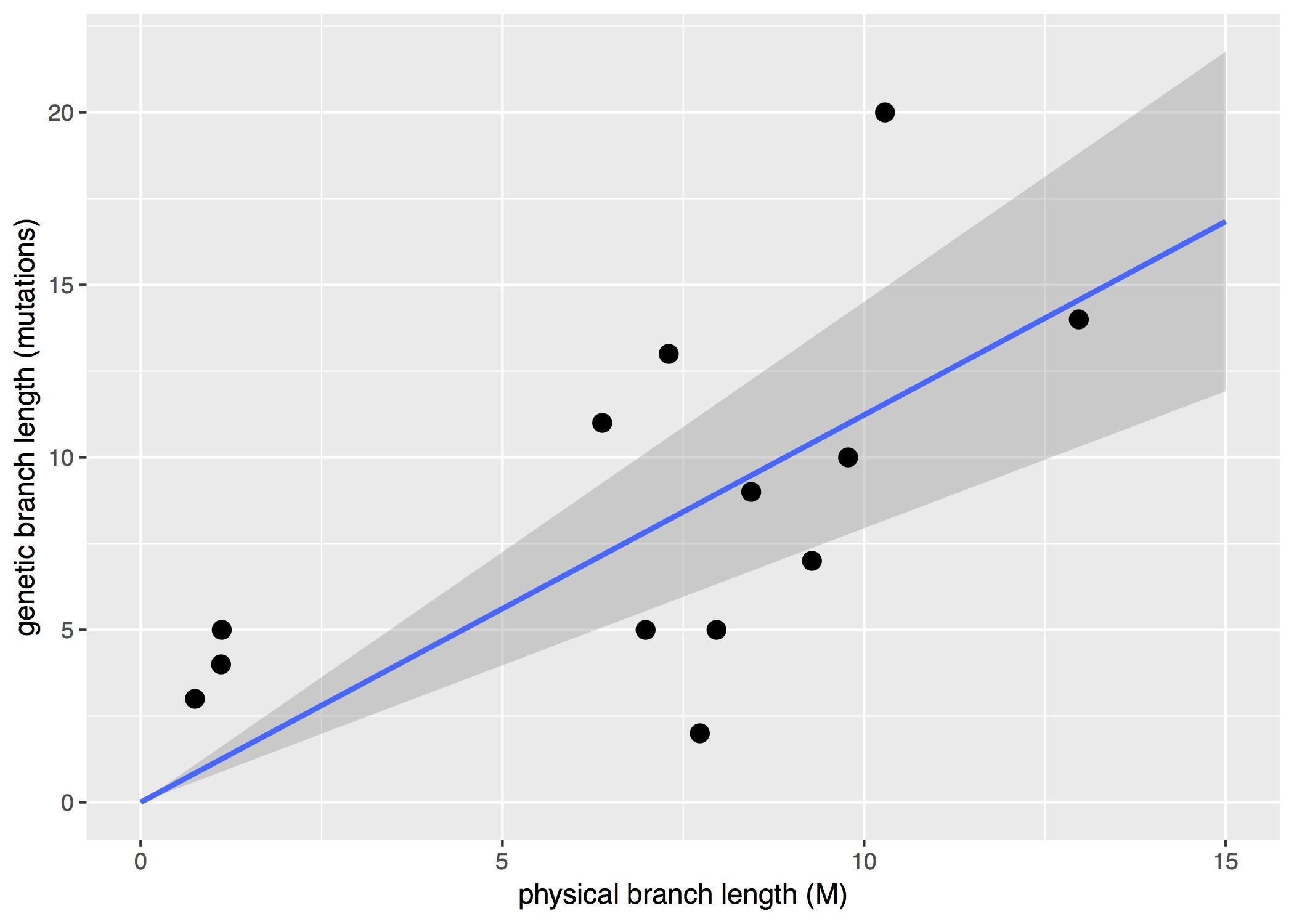


**Supplementary figure 4.1**: the relationship between physical branch length and genetic branch length. The line of best fit is calculated from a regression forced through the origin.


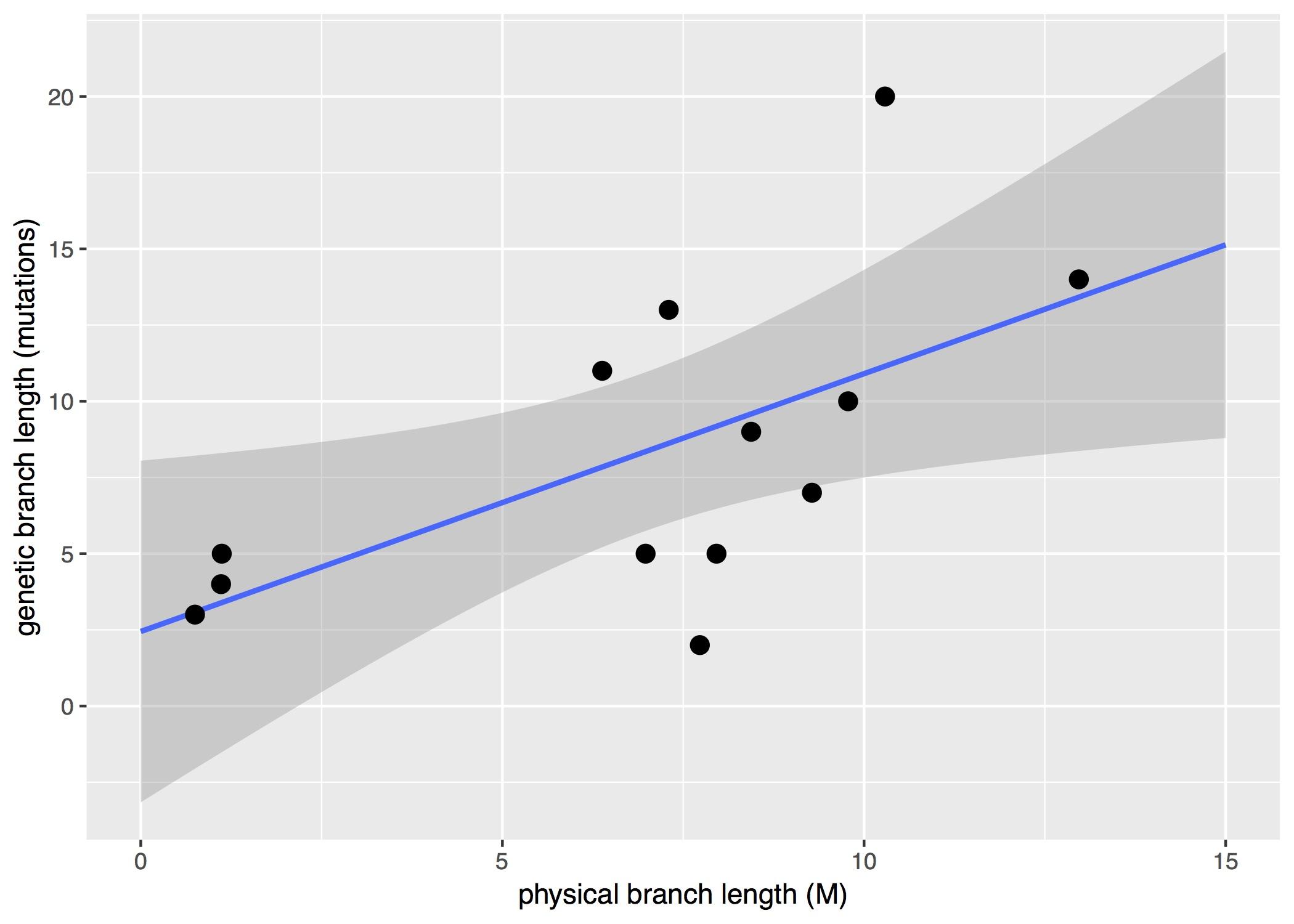


**Supplementary figure 4.2**: the relationship between physical branch length and genetic branch length. The line of best fit is calculated from a regression that was not forced through the origin.

**5. Effect of Filtering on the Number of Variants**

Table 5.1 shows the number of variants remaining after each filtering step in the “Variant Calling for Positive Control” section of the main manuscript. Note that the filters are applied sequentially, such that each row in the table implies that the filter in that row, in addition to the filters in all preceding rows, have been applied. We do not include an estimate of the False Discovery Rate here, because calculating it requires the estimation of a denominator that represents a number of inferred mutations. All of the numbers prior to the application of the final filter (in which we only include variable sites) include both constant heterozygous sites as well as sites that vary among the 8 branches of the tree, meaning that none of them (except the one in the final row) can be used to calculate a meaningful False Discovery Rate.

The table shows, as expected, that the application of each new filter reduces the total number of sites being considered, which in turn increases the false negative rate and decreases the number of false positive mutations that we detect.

| Supplementary table 5.1: the effect of different filters on the number of sites remaining under consideration. | | | |
| --- | --- | --- | --- |
| **Filter** | **Total Sites** | **False Negative Rate** | **Number of false positive mutations** |
| GATK output on 11 scaffolds | 9679544 | 55.4% | 1033.18 |
| Site depth <= 500 | 9109287 | 56.1% | 918.39 |
| ExcessHet <= 40 | 4958664 | 56.3% | 164.86 |
| Not within 50bp of an indel | 3245141 | 62.0% | 99.71 |
| Biallelic SNPs | 2049795 | 62.2% | 92.35 |
| Outside repeats | 967888 | 70.1% | 55.71 |
| All 3 replicates match | 65949 | 77.3% | 55.71 |
| Only include variable sites | 99 | 77.3% | 55.71 |
